# Supplementary material for: Mapping the evolution of fertility support policies in China: A content and instrumental analysis
Source: PLoS One. 2025 Oct 9;20(10):e0332137. doi: 10.1371/journal.pone.0332137 (PMC12510515; doi:10.1371/journal.pone.0332137)
Supplement: S1 Appendix — (ZIP) [file pone.0332137.s001.zip › S1 Appendix. 226 original policy documents/143-中共中央关于制定国民经济和社会发展第十四个五年规划和二〇三五年远景目标的建议(FBM-CLI-16-347489).doc]

中共中央关于制定国民经济和社会发展第十四个五年规划和二〇三五年远景目标的建议

**发布部门：** [中国共产党中央委员会](https://www.pkulaw.com/law/chl?Aggs.IssueDepartment=a01)

**发布日期：**2020.10.29

**实施日期：**2020.10.29

**时效性：** [现行有效](https://www.pkulaw.com/law/chl?Aggs.TimelinessDic=01)

**效力级别：** [党内法规制度](https://www.pkulaw.com/law/chl?Aggs.EffectivenessDic=XR12)

**法规类别：** [机关工作综合规定](https://www.pkulaw.com/law/chl?Aggs.Category=00301)

**专题分类：** [依法治国](https://www.pkulaw.com/law/chl?Aggs.SpecialType=019)

中共中央关于制定国民经济和社会发展第十四个五年规划和二〇三五年远景目标的建议
（2020年10月29日中国共产党第十九届中央委员会第五次全体会议通过）

　　“十四五”时期是我国全面建成小康社会、实现第一个百年奋斗目标之后，乘势而上开启全面建设社会主义现代化国家新征程、向第二个百年奋斗目标进军的第一个五年。中国共产党第十九届中央委员会第五次全体会议深入分析国际国内形势，就制定国民经济和社会发展“十四五”规划和二〇三五年远景目标提出以下建议。
　　一、全面建成小康社会，开启全面建设社会主义现代化国家新征程
　　1.决胜全面建成小康社会取得决定性成就。“十三五”时期是全面建成小康社会决胜阶段。面对错综复杂的国际形势、艰巨繁重的国内改革发展稳定任务特别是新冠肺炎疫情严重冲击，以习近平同志为核心的党中央不忘初心、牢记使命，团结带领全党全国各族人民砥砺前行、开拓创新，奋发有为推进党和国家各项事业。全面深化改革取得重大突破，全面依法治国取得重大进展，全面从严治党取得重大成果，国家治理体系和治理能力现代化加快推进，中国共产党领导和我国社会主义制度优势进一步彰显；经济实力、科技实力、综合国力跃上新的大台阶，经济运行总体平稳，经济结构持续优化，预计二〇二〇年国内生产总值突破一百万亿元；脱贫攻坚成果举世瞩目，五千五百七十五万农村贫困人口实现脱贫；粮食年产量连续五年稳定在一万三千亿斤以上；污染防治力度加大，生态环境明显改善；对外开放持续扩大，共建“一带一路”成果丰硕；人民生活水平显著提高，高等教育进入普及化阶段，城镇新增就业超过六千万人，建成世界上规模最大的社会保障体系，基本医疗保险覆盖超过十三亿人，基本养老保险覆盖近十亿人，新冠肺炎疫情防控取得重大战略成果；文化事业和文化产业繁荣发展；国防和军队建设水平大幅提升，军队组织形态实现重大变革；国家安全全面加强，社会保持和谐稳定。“十三五”规划目标任务即将完成，全面建成小康社会胜利在望，中华民族伟大复兴向前迈出了新的一大步，社会主义中国以更加雄伟的身姿屹立于世界东方。全党全国各族人民要再接再厉、一鼓作气，确保如期打赢脱贫攻坚战，确保如期全面建成小康社会、实现第一个百年奋斗目标，为开启全面建设社会主义现代化国家新征程奠定坚实基础。

　　2.我国发展环境面临深刻复杂变化。当前和今后一个时期，我国发展仍然处于重要战略机遇期，但机遇和挑战都有新的发展变化。当今世界正经历百年未有之大变局，新一轮科技革命和产业变革深入发展，国际力量对比深刻调整，和平与发展仍然是时代主题，人类命运共同体理念深入人心，同时国际环境日趋复杂，不稳定性不确定性明显增加，新冠肺炎疫情影响广泛深远，经济全球化遭遇逆流，世界进入动荡变革期，单边主义、保护主义、霸权主义对世界和平与发展构成威胁。我国已转向高质量发展阶段，制度优势显著，治理效能提升，经济长期向好，物质基础雄厚，人力资源丰富，市场空间广阔，发展韧性强劲，社会大局稳定，继续发展具有多方面优势和条件，同时我国发展不平衡不充分问题仍然突出，重点领域关键环节改革任务仍然艰巨，创新能力不适应高质量发展要求，农业基础还不稳固，城乡区域发展和收入分配差距较大，生态环保任重道远，民生保障存在短板，社会治理还有弱项。全党要统筹中华民族伟大复兴战略全局和世界百年未有之大变局，深刻认识我国社会主要矛盾变化带来的新特征新要求，深刻认识错综复杂的国际环境带来的新矛盾新挑战，增强机遇意识和风险意识，立足社会主义初级阶段基本国情，保持战略定力，办好自己的事，认识和把握发展规律，发扬斗争精神，树立底线思维，准确识变、科学应变、主动求变，善于在危机中育先机、于变局中开新局，抓住机遇，应对挑战，趋利避害，奋勇前进。

　　3.到二〇三五年基本实现社会主义现代化远景目标。党的十九大对实现第二个百年奋斗目标作出分两个阶段推进的战略安排，即到二〇三五年基本实现社会主义现代化，到本世纪中叶把我国建成富强民主文明和谐美丽的社会主义现代化强国。展望二〇三五年，我国经济实力、科技实力、综合国力将大幅跃升，经济总量和城乡居民人均收入将再迈上新的大台阶，关键核心技术实现重大突破，进入创新型国家前列；基本实现新型工业化、信息化、城镇化、农业现代化，建成现代化经济体系；基本实现国家治理体系和治理能力现代化，人民平等参与、平等发展权利得到充分保障，基本建成法治国家、法治政府、法治社会；建成文化强国、教育强国、人才强国、体育强国、健康中国，国民素质和社会文明程度达到新高度，国家文化软实力显著增强；广泛形成绿色生产生活方式，碳排放达峰后稳中有降，生态环境根本好转，美丽中国建设目标基本实现；形成对外开放新格局，参与国际经济合作和竞争新优势明显增强；人均国内生产总值达到中等发达国家水平，中等收入群体显著扩大，基本公共服务实现均等化，城乡区域发展差距和居民生活水平差距显著缩小；平安中国建设达到更高水平，基本实现国防和军队现代化；人民生活更加美好，人的全面发展、全体人民共同富裕取得更为明显的实质性进展。
　　二、“十四五”时期经济社会发展指导方针和主要目标

　　4.“十四五”时期经济社会发展指导思想。高举中国特色社会主义伟大旗帜，深入贯彻党的十九大和十九届二中、三中、四中、五中全会精神，坚持以马克思列宁主义、毛泽东思想、邓小平理论、“三个代表”重要思想、科学发展观、习近平新时代中国特色社会主义思想为指导，全面贯彻党的基本理论、基本路线、基本方略，统筹推进经济建设、政治建设、文化建设、社会建设、生态文明建设的总体布局，协调推进全面建设社会主义现代化国家、全面深化改革、全面依法治国、全面从严治党的战略布局，坚定不移贯彻创新、协调、绿色、开放、共享的新发展理念，坚持稳中求进工作总基调，以推动高质量发展为主题，以深化供给侧结构性改革为主线，以改革创新为根本动力，以满足人民日益增长的美好生活需要为根本目的，统筹发展和安全，加快建设现代化经济体系，加快构建以国内大循环为主体、国内国际双循环相互促进的新发展格局，推进国家治理体系和治理能力现代化，实现经济行稳致远、社会安定和谐，为全面建设社会主义现代化国家开好局、起好步。

　　5.“十四五”时期经济社会发展必须遵循的原则。
　　--坚持党的全面领导。坚持和完善党领导经济社会发展的体制机制，坚持和完善中国特色社会主义制度，不断提高贯彻新发展理念、构建新发展格局能力和水平，为实现高质量发展提供根本保证。
　　--坚持以人民为中心。坚持人民主体地位，坚持共同富裕方向，始终做到发展为了人民、发展依靠人民、发展成果由人民共享，维护人民根本利益，激发全体人民积极性、主动性、创造性，促进社会公平，增进民生福祉，不断实现人民对美好生活的向往。
　　--坚持新发展理念。把新发展理念贯穿发展全过程和各领域，构建新发展格局，切实转变发展方式，推动质量变革、效率变革、动力变革，实现更高质量、更有效率、更加公平、更可持续、更为安全的发展。
　　--坚持深化改革开放。坚定不移推进改革，坚定不移扩大开放，加强国家治理体系和治理能力现代化建设，破除制约高质量发展、高品质生活的体制机制障碍，强化有利于提高资源配置效率、有利于调动全社会积极性的重大改革开放举措，持续增强发展动力和活力。
　　--坚持系统观念。加强前瞻性思考、全局性谋划、战略性布局、整体性推进，统筹国内国际两个大局，办好发展安全两件大事，坚持全国一盘棋，更好发挥中央、地方和各方面积极性，着力固根基、扬优势、补短板、强弱项，注重防范化解重大风险挑战，实现发展质量、结构、规模、速度、效益、安全相统一。

　　6.“十四五”时期经济社会发展主要目标。锚定二〇三五年远景目标，综合考虑国内外发展趋势和我国发展条件，坚持目标导向和问题导向相结合，坚持守正和创新相统一，今后五年经济社会发展要努力实现以下主要目标。
　　--经济发展取得新成效。发展是解决我国一切问题的基础和关键，发展必须坚持新发展理念，在质量效益明显提升的基础上实现经济持续健康发展，增长潜力充分发挥，国内市场更加强大，经济结构更加优化，创新能力显著提升，产业基础高级化、产业链现代化水平明显提高，农业基础更加稳固，城乡区域发展协调性明显增强，现代化经济体系建设取得重大进展。
　　--改革开放迈出新步伐。社会主义市场经济体制更加完善，高标准市场体系基本建成，市场主体更加充满活力，产权制度改革和要素市场化配置改革取得重大进展，公平竞争制度更加健全，更高水平开放型经济新体制基本形成。
　　--社会文明程度得到新提高。社会主义核心价值观深入人心，人民思想道德素质、科学文化素质和身心健康素质明显提高，公共文化服务体系和文化产业体系更加健全，人民精神文化生活日益丰富，中华文化影响力进一步提升，中华民族凝聚力进一步增强。
　　--生态文明建设实现新进步。国土空间开发保护格局得到优化，生产生活方式绿色转型成效显著，能源资源配置更加合理、利用效率大幅提高，主要污染物排放总量持续减少，生态环境持续改善，生态安全屏障更加牢固，城乡人居环境明显改善。
　　--民生福祉达到新水平。实现更加充分更高质量就业，居民收入增长和经济增长基本同步，分配结构明显改善，基本公共服务均等化水平明显提高，全民受教育程度不断提升，多层次社会保障体系更加健全，卫生健康体系更加完善，脱贫攻坚成果巩固拓展，乡村振兴战略全面推进。
　　--国家治理效能得到新提升。社会主义民主法治更加健全，社会公平正义进一步彰显，国家行政体系更加完善，政府作用更好发挥，行政效率和公信力显著提升，社会治理特别是基层治理水平明显提高，防范化解重大风险体制机制不断健全，突发公共事件应急能力显著增强，自然灾害防御水平明显提升，发展安全保障更加有力，国防和军队现代化迈出重大步伐。
　　三、坚持创新驱动发展，全面塑造发展新优势
　　坚持创新在我国现代化建设全局中的核心地位，把科技自立自强作为国家发展的战略支撑，面向世界科技前沿、面向经济主战场、面向国家重大需求、面向人民生命健康，深入实施科教兴国战略、人才强国战略、创新驱动发展战略，完善国家创新体系，加快建设科技强国。

　　7.强化国家战略科技力量。制定科技强国行动纲要，健全社会主义市场经济条件下新型举国体制，打好关键核心技术攻坚战，提高创新链整体效能。加强基础研究、注重原始创新，优化学科布局和研发布局，推进学科交叉融合，完善共性基础技术供给体系。瞄准人工智能、量子信息、集成电路、生命健康、脑科学、生物育种、空天科技、深地深海等前沿领域，实施一批具有前瞻性、战略性的国家重大科技项目。制定实施战略性科学计划和科学工程，推进科研院所、高校、企业科研力量优化配置和资源共享。推进国家实验室建设，重组国家重点实验室体系。布局建设综合性国家科学中心和区域性创新高地，支持北京、上海、粤港澳大湾区形成国际科技创新中心。构建国家科研论文和科技信息高端交流平台。

　　8.提升企业技术创新能力。强化企业创新主体地位，促进各类创新要素向企业集聚。推进产学研深度融合，支持企业牵头组建创新联合体，承担国家重大科技项目。发挥企业家在技术创新中的重要作用，鼓励企业加大研发投入，对企业投入基础研究实行税收优惠。发挥大企业引领支撑作用，支持创新型中小微企业成长为创新重要发源地，加强共性技术平台建设，推动产业链上中下游、大中小企业融通创新。

　　9.激发人才创新活力。贯彻尊重劳动、尊重知识、尊重人才、尊重创造方针，深化人才发展体制机制改革，全方位培养、引进、用好人才，造就更多国际一流的科技领军人才和创新团队，培养具有国际竞争力的青年科技人才后备军。健全以创新能力、质量、实效、贡献为导向的科技人才评价体系。加强学风建设，坚守学术诚信。深化院士制度改革。健全创新激励和保障机制，构建充分体现知识、技术等创新要素价值的收益分配机制，完善科研人员职务发明成果权益分享机制。加强创新型、应用型、技能型人才培养，实施知识更新工程、技能提升行动，壮大高水平工程师和高技能人才队伍。支持发展高水平研究型大学，加强基础研究人才培养。实行更加开放的人才政策，构筑集聚国内外优秀人才的科研创新高地。

　　10.完善科技创新体制机制。深入推进科技体制改革，完善国家科技治理体系，优化国家科技规划体系和运行机制，推动重点领域项目、基地、人才、资金一体化配置。改进科技项目组织管理方式，实行“揭榜挂帅”等制度。完善科技评价机制，优化科技奖励项目。加快科研院所改革，扩大科研自主权。加强知识产权保护，大幅提高科技成果转移转化成效。加大研发投入，健全政府投入为主、社会多渠道投入机制，加大对基础前沿研究支持。完善金融支持创新体系，促进新技术产业化规模化应用。弘扬科学精神和工匠精神，加强科普工作，营造崇尚创新的社会氛围。健全科技伦理体系。促进科技开放合作，研究设立面向全球的科学研究基金。
　　四、加快发展现代产业体系，推动经济体系优化升级
　　坚持把发展经济着力点放在实体经济上，坚定不移建设制造强国、质量强国、网络强国、数字中国，推进产业基础高级化、产业链现代化，提高经济质量效益和核心竞争力。

　　11.提升产业链供应链现代化水平。保持制造业比重基本稳定，巩固壮大实体经济根基。坚持自主可控、安全高效，分行业做好供应链战略设计和精准施策，推动全产业链优化升级。锻造产业链供应链长板，立足我国产业规模优势、配套优势和部分领域先发优势，打造新兴产业链，推动传统产业高端化、智能化、绿色化，发展服务型制造。完善国家质量基础设施，加强标准、计量、专利等体系和能力建设，深入开展质量提升行动。促进产业在国内有序转移，优化区域产业链布局，支持老工业基地转型发展。补齐产业链供应链短板，实施产业基础再造工程，加大重要产品和关键核心技术攻关力度，发展先进适用技术，推动产业链供应链多元化。优化产业链供应链发展环境，强化要素支撑。加强国际产业安全合作，形成具有更强创新力、更高附加值、更安全可靠的产业链供应链。

　　12.发展战略性新兴产业。加快壮大新一代信息技术、生物技术、新能源、新材料、高端装备、新能源汽车、绿色环保以及航空航天、海洋装备等产业。推动互联网、大数据、人工智能等同各产业深度融合，推动先进制造业集群发展，构建一批各具特色、优势互补、结构合理的战略性新兴产业增长引擎，培育新技术、新产品、新业态、新模式。促进平台经济、共享经济健康发展。鼓励企业兼并重组，防止低水平重复建设。

　　13.加快发展现代服务业。推动生产性服务业向专业化和价值链高端延伸，推动各类市场主体参与服务供给，加快发展研发设计、现代物流、法律服务等服务业，推动现代服务业同先进制造业、现代农业深度融合，加快推进服务业数字化。推动生活性服务业向高品质和多样化升级，加快发展健康、养老、育幼、文化、旅游、体育、家政、物业等服务业，加强公益性、基础性服务业供给。推进服务业标准化、品牌化建设。

　　14.统筹推进基础设施建设。构建系统完备、高效实用、智能绿色、安全可靠的现代化基础设施体系。系统布局新型基础设施，加快第五代移动通信、工业互联网、大数据中心等建设。加快建设交通强国，完善综合运输大通道、综合交通枢纽和物流网络，加快城市群和都市圈轨道交通网络化，提高农村和边境地区交通通达深度。推进能源革命，完善能源产供储销体系，加强国内油气勘探开发，加快油气储备设施建设，加快全国干线油气管道建设，建设智慧能源系统，优化电力生产和输送通道布局，提升新能源消纳和存储能力，提升向边远地区输配电能力。加强水利基础设施建设，提升水资源优化配置和水旱灾害防御能力。

　　15.加快数字化发展。发展数字经济，推进数字产业化和产业数字化，推动数字经济和实体经济深度融合，打造具有国际竞争力的数字产业集群。加强数字社会、数字政府建设，提升公共服务、社会治理等数字化智能化水平。建立数据资源产权、交易流通、跨境传输和安全保护等基础制度和标准规范，推动数据资源开发利用。扩大基础公共信息数据有序开放，建设国家数据统一共享开放平台。保障国家数据安全，加强个人信息保护。提升全民数字技能，实现信息服务全覆盖。积极参与数字领域国际规则和标准制定。
　　五、形成强大国内市场，构建新发展格局
　　坚持扩大内需这个战略基点，加快培育完整内需体系，把实施扩大内需战略同深化供给侧结构性改革有机结合起来，以创新驱动、高质量供给引领和创造新需求。

　　16.畅通国内大循环。依托强大国内市场，贯通生产、分配、流通、消费各环节，打破行业垄断和地方保护，形成国民经济良性循环。优化供给结构，改善供给质量，提升供给体系对国内需求的适配性。推动金融、房地产同实体经济均衡发展，实现上下游、产供销有效衔接，促进农业、制造业、服务业、能源资源等产业门类关系协调。破除妨碍生产要素市场化配置和商品服务流通的体制机制障碍，降低全社会交易成本。完善扩大内需的政策支撑体系，形成需求牵引供给、供给创造需求的更高水平动态平衡。

　　17.促进国内国际双循环。立足国内大循环，发挥比较优势，协同推进强大国内市场和贸易强国建设，以国内大循环吸引全球资源要素，充分利用国内国际两个市场两种资源，积极促进内需和外需、进口和出口、引进外资和对外投资协调发展，促进国际收支基本平衡。完善内外贸一体化调控体系，促进内外贸法律法规、监管体制、经营资质、质量标准、检验检疫、认证认可等相衔接，推进同线同标同质。优化国内国际市场布局、商品结构、贸易方式，提升出口质量，增加优质产品进口，实施贸易投资融合工程，构建现代物流体系。

　　18.全面促进消费。增强消费对经济发展的基础性作用，顺应消费升级趋势，提升传统消费，培育新型消费，适当增加公共消费。以质量品牌为重点，促进消费向绿色、健康、安全发展，鼓励消费新模式新业态发展。推动汽车等消费品由购买管理向使用管理转变，促进住房消费健康发展。健全现代流通体系，发展无接触交易服务，降低企业流通成本，促进线上线下消费融合发展，开拓城乡消费市场。发展服务消费，放宽服务消费领域市场准入。完善节假日制度，落实带薪休假制度，扩大节假日消费。培育国际消费中心城市。改善消费环境，强化消费者权益保护。

　　19.拓展投资空间。优化投资结构，保持投资合理增长，发挥投资对优化供给结构的关键作用。加快补齐基础设施、市政工程、农业农村、公共安全、生态环保、公共卫生、物资储备、防灾减灾、民生保障等领域短板，推动企业设备更新和技术改造，扩大战略性新兴产业投资。推进新型基础设施、新型城镇化、交通水利等重大工程建设，支持有利于城乡区域协调发展的重大项目建设。实施川藏铁路、西部陆海新通道、国家水网、雅鲁藏布江下游水电开发、星际探测、北斗产业化等重大工程，推进重大科研设施、重大生态系统保护修复、公共卫生应急保障、重大引调水、防洪减灾、送电输气、沿边沿江沿海交通等一批强基础、增功能、利长远的重大项目建设。发挥政府投资撬动作用，激发民间投资活力，形成市场主导的投资内生增长机制。
　　六、全面深化改革，构建高水平社会主义市场经济体制
　　坚持和完善社会主义基本经济制度，充分发挥市场在资源配置中的决定性作用，更好发挥政府作用，推动有效市场和有为政府更好结合。

　　20.激发各类市场主体活力。毫不动摇巩固和发展公有制经济，毫不动摇鼓励、支持、引导非公有制经济发展。深化国资国企改革，做强做优做大国有资本和国有企业。加快国有经济布局优化和结构调整，发挥国有经济战略支撑作用。加快完善中国特色现代企业制度，深化国有企业混合所有制改革。健全管资本为主的国有资产监管体制，深化国有资本投资、运营公司改革。推进能源、铁路、电信、公用事业等行业竞争性环节市场化改革。优化民营经济发展环境，构建亲清政商关系，促进非公有制经济健康发展和非公有制经济人士健康成长，依法平等保护民营企业产权和企业家权益，破除制约民营企业发展的各种壁垒，完善促进中小微企业和个体工商户发展的法律环境和政策体系。弘扬企业家精神，加快建设世界一流企业。

　　21.完善宏观经济治理。健全以国家发展规划为战略导向，以财政政策和货币政策为主要手段，就业、产业、投资、消费、环保、区域等政策紧密配合，目标优化、分工合理、高效协同的宏观经济治理体系。完善宏观经济政策制定和执行机制，重视预期管理，提高调控的科学性。加强国际宏观经济政策协调，搞好跨周期政策设计，提高逆周期调节能力，促进经济总量平衡、结构优化、内外均衡。加强宏观经济治理数据库等建设，提升大数据等现代技术手段辅助治理能力。推进统计现代化改革。

　　22.建立现代财税金融体制。加强财政资源统筹，加强中期财政规划管理，增强国家重大战略任务财力保障。深化预算管理制度改革，强化对预算编制的宏观指导。推进财政支出标准化，强化预算约束和绩效管理。明确中央和地方政府事权与支出责任，健全省以下财政体制，增强基层公共服务保障能力。完善现代税收制度，健全地方税、直接税体系，优化税制结构，适当提高直接税比重，深化税收征管制度改革。健全政府债务管理制度。建设现代中央银行制度，完善货币供应调控机制，稳妥推进数字货币研发，健全市场化利率形成和传导机制。构建金融有效支持实体经济的体制机制，提升金融科技水平，增强金融普惠性。深化国有商业银行改革，支持中小银行和农村信用社持续健康发展，改革优化政策性金融。全面实行股票发行注册制，建立常态化退市机制，提高直接融资比重。推进金融双向开放。完善现代金融监管体系，提高金融监管透明度和法治化水平，完善存款保险制度，健全金融风险预防、预警、处置、问责制度体系，对违法违规行为零容忍。

　　23.建设高标准市场体系。健全市场体系基础制度，坚持平等准入、公正监管、开放有序、诚信守法，形成高效规范、公平竞争的国内统一市场。实施高标准市场体系建设行动。健全产权执法司法保护制度。实施统一的[市场准入负面清单](https://www.pkulaw.com/chl/fb62cc1e71e4ee0bbdfb.html)制度。继续放宽准入限制。健全公平竞争审查机制，加强反垄断和反不正当竞争执法司法，提升市场综合监管能力。深化土地管理制度改革。推进土地、劳动力、资本、技术、数据等要素市场化改革。健全要素市场运行机制，完善要素交易规则和服务体系。

　　24.加快转变政府职能。建设职责明确、依法行政的政府治理体系。深化简政放权、放管结合、优化服务改革，全面实行政府权责清单制度。持续优化市场化法治化国际化营商环境。实施涉企经营许可事项清单管理，加强事中事后监管，对新产业新业态实行包容审慎监管。健全重大政策事前评估和事后评价制度，畅通参与政策制定的渠道，提高决策科学化、民主化、法治化水平。推进政务服务标准化、规范化、便利化，深化政务公开。深化行业协会、商会和中介机构改革。
　　七、优先发展农业农村，全面推进乡村振兴
　　坚持把解决好“三农”问题作为全党工作重中之重，走中国特色社会主义乡村振兴道路，全面实施乡村振兴战略，强化以工补农、以城带乡，推动形成工农互促、城乡互补、协调发展、共同繁荣的新型工农城乡关系，加快农业农村现代化。

　　25.提高农业质量效益和竞争力。适应确保国计民生要求，以保障国家粮食安全为底线，健全农业支持保护制度。坚持最严格的耕地保护制度，深入实施藏粮于地、藏粮于技战略，加大农业水利设施建设力度，实施高标准农田建设工程，强化农业科技和装备支撑，提高农业良种化水平，健全动物防疫和农作物病虫害防治体系，建设智慧农业。强化绿色导向、标准引领和质量安全监管，建设农业现代化示范区。推动农业供给侧结构性改革，优化农业生产结构和区域布局，加强粮食生产功能区、重要农产品生产保护区和特色农产品优势区建设，推进优质粮食工程。完善粮食主产区利益补偿机制。保障粮、棉、油、糖、肉等重要农产品供给安全，提升收储调控能力。开展粮食节约行动。发展县域经济，推动农村一二三产业融合发展，丰富乡村经济业态，拓展农民增收空间。

　　26.实施乡村建设行动。把乡村建设摆在社会主义现代化建设的重要位置。强化县城综合服务能力，把乡镇建成服务农民的区域中心。统筹县域城镇和村庄规划建设，保护传统村落和乡村风貌。完善乡村水、电、路、气、通信、广播电视、物流等基础设施，提升农房建设质量。因地制宜推进农村改厕、生活垃圾处理和污水治理，实施河湖水系综合整治，改善农村人居环境。提高农民科技文化素质，推动乡村人才振兴。

　　27.深化农村改革。健全城乡融合发展机制，推动城乡要素平等交换、双向流动，增强农业农村发展活力。落实第二轮土地承包到期后再延长三十年政策，加快培育农民合作社、家庭农场等新型农业经营主体，健全农业专业化社会化服务体系，发展多种形式适度规模经营，实现小农户和现代农业有机衔接。健全城乡统一的建设用地市场，积极探索实施农村集体经营性建设用地入市制度。建立土地征收公共利益用地认定机制，缩小土地征收范围。探索宅基地所有权、资格权、使用权分置实现形式。保障进城落户农民土地承包权、宅基地使用权、集体收益分配权，鼓励依法自愿有偿转让。深化农村集体产权制度改革，发展新型农村集体经济。健全农村金融服务体系，发展农业保险。

　　28.实现巩固拓展脱贫攻坚成果同乡村振兴有效衔接。建立农村低收入人口和欠发达地区帮扶机制，保持财政投入力度总体稳定，接续推进脱贫地区发展。健全防止返贫监测和帮扶机制，做好易地扶贫搬迁后续帮扶工作，加强扶贫项目资金资产管理和监督，推动特色产业可持续发展。健全农村社会保障和救助制度。在西部地区脱贫县中集中支持一批乡村振兴重点帮扶县，增强其巩固脱贫成果及内生发展能力。坚持和完善东西部协作和对口支援、社会力量参与帮扶等机制。
　　八、优化国土空间布局，推进区域协调发展和新型城镇化
　　坚持实施区域重大战略、区域协调发展战略、主体功能区战略，健全区域协调发展体制机制，完善新型城镇化战略，构建高质量发展的国土空间布局和支撑体系。

　　29.构建国土空间开发保护新格局。立足资源环境承载能力，发挥各地比较优势，逐步形成城市化地区、农产品主产区、生态功能区三大空间格局，优化重大基础设施、重大生产力和公共资源布局。支持城市化地区高效集聚经济和人口、保护基本农田和生态空间，支持农产品主产区增强农业生产能力，支持生态功能区把发展重点放到保护生态环境、提供生态产品上，支持生态功能区的人口逐步有序转移，形成主体功能明显、优势互补、高质量发展的国土空间开发保护新格局。

　　30.推动区域协调发展。推动西部大开发形成新格局，推动东北振兴取得新突破，促进中部地区加快崛起，鼓励东部地区加快推进现代化。支持革命老区、民族地区加快发展，加强边疆地区建设，推进兴边富民、稳边固边。推进京津冀协同发展、长江经济带发展、粤港澳大湾区建设、长三角一体化发展，打造创新平台和新增长极。推动黄河流域生态保护和高质量发展。高标准、高质量建设雄安新区。坚持陆海统筹，发展海洋经济，建设海洋强国。健全区域战略统筹、市场一体化发展、区域合作互助、区际利益补偿等机制，更好促进发达地区和欠发达地区、东中西部和东北地区共同发展。完善转移支付制度，加大对欠发达地区财力支持，逐步实现基本公共服务均等化。

　　31.推进以人为核心的新型城镇化。实施城市更新行动，推进城市生态修复、功能完善工程，统筹城市规划、建设、管理，合理确定城市规模、人口密度、空间结构，促进大中小城市和小城镇协调发展。强化历史文化保护、塑造城市风貌，加强城镇老旧小区改造和社区建设，增强城市防洪排涝能力，建设海绵城市、韧性城市。提高城市治理水平，加强特大城市治理中的风险防控。坚持房子是用来住的、不是用来炒的定位，租购并举、因城施策，促进房地产市场平稳健康发展。有效增加保障性住房供给，完善土地出让收入分配机制，探索支持利用集体建设用地按照规划建设租赁住房，完善长租房政策，扩大保障性租赁住房供给。深化户籍制度改革，完善财政转移支付和城镇新增建设用地规模与农业转移人口市民化挂钩政策，强化基本公共服务保障，加快农业转移人口市民化。优化行政区划设置，发挥中心城市和城市群带动作用，建设现代化都市圈。推进成渝地区双城经济圈建设。推进以县城为重要载体的城镇化建设。
　　九、繁荣发展文化事业和文化产业，提高国家文化软实力
　　坚持马克思主义在意识形态领域的指导地位，坚定文化自信，坚持以社会主义核心价值观引领文化建设，加强社会主义精神文明建设，围绕举旗帜、聚民心、育新人、兴文化、展形象的使命任务，促进满足人民文化需求和增强人民精神力量相统一，推进社会主义文化强国建设。

　　32.提高社会文明程度。推动形成适应新时代要求的思想观念、精神面貌、文明风尚、行为规范。深入开展习近平新时代中国特色社会主义思想学习教育，推进马克思主义理论研究和建设工程。推动理想信念教育常态化制度化，加强党史、新中国史、改革开放史、社会主义发展史教育，加强爱国主义、集体主义、社会主义教育，弘扬党和人民在各个历史时期奋斗中形成的伟大精神，推进公民道德建设，实施文明创建工程，拓展新时代文明实践中心建设。健全志愿服务体系，广泛开展志愿服务关爱行动。弘扬诚信文化，推进诚信建设。提倡艰苦奋斗、勤俭节约，开展以劳动创造幸福为主题的宣传教育。加强家庭、家教、家风建设。加强网络文明建设，发展积极健康的网络文化。

　　33.提升公共文化服务水平。全面繁荣新闻出版、广播影视、文学艺术、哲学社会科学事业。实施文艺作品质量提升工程，加强现实题材创作生产，不断推出反映时代新气象、讴歌人民新创造的文艺精品。推进媒体深度融合，实施全媒体传播工程，做强新型主流媒体，建强用好县级融媒体中心。推进城乡公共文化服务体系一体建设，创新实施文化惠民工程，广泛开展群众性文化活动，推动公共文化数字化建设。加强国家重大文化设施和文化项目建设，推进国家版本馆、国家文献储备库、智慧广电等工程。传承弘扬中华优秀传统文化，加强文物古籍保护、研究、利用，强化重要文化和自然遗产、非物质文化遗产系统性保护，加强各民族优秀传统手工艺保护和传承，建设长城、大运河、长征、黄河等国家文化公园。广泛开展全民健身运动，增强人民体质。筹办好北京冬奥会、冬残奥会。

　　34.健全现代文化产业体系。坚持把社会效益放在首位、社会效益和经济效益相统一，深化文化体制改革，完善文化产业规划和政策，加强文化市场体系建设，扩大优质文化产品供给。实施文化产业数字化战略，加快发展新型文化企业、文化业态、文化消费模式。规范发展文化产业园区，推动区域文化产业带建设。推动文化和旅游融合发展，建设一批富有文化底蕴的世界级旅游景区和度假区，打造一批文化特色鲜明的国家级旅游休闲城市和街区，发展红色旅游和乡村旅游。以讲好中国故事为着力点，创新推进国际传播，加强对外文化交流和多层次文明对话。
　　十、推动绿色发展，促进人与自然和谐共生
　　坚持绿水青山就是金山银山理念，坚持尊重自然、顺应自然、保护自然，坚持节约优先、保护优先、自然恢复为主，守住自然生态安全边界。深入实施可持续发展战略，完善生态文明领域统筹协调机制，构建生态文明体系，促进经济社会发展全面绿色转型，建设人与自然和谐共生的现代化。

　　35.加快推动绿色低碳发展。强化国土空间规划和用途管控，落实生态保护、基本农田、城镇开发等空间管控边界，减少人类活动对自然空间的占用。强化绿色发展的法律和政策保障，发展绿色金融，支持绿色技术创新，推进清洁生产，发展环保产业，推进重点行业和重要领域绿色化改造。推动能源清洁低碳安全高效利用。发展绿色建筑。开展绿色生活创建活动。降低碳排放强度，支持有条件的地方率先达到碳排放峰值，制定二〇三〇年前碳排放达峰行动方案。

　　36.持续改善环境质量。增强全社会生态环保意识，深入打好污染防治攻坚战。继续开展污染防治行动，建立地上地下、陆海统筹的生态环境治理制度。强化多污染物协同控制和区域协同治理，加强细颗粒物和臭氧协同控制，基本消除重污染天气。治理城乡生活环境，推进城镇污水管网全覆盖，基本消除城市黑臭水体。推进化肥农药减量化和土壤污染治理，加强白色污染治理。加强危险废物医疗废物收集处理。完成重点地区危险化学品生产企业搬迁改造。重视新污染物治理。全面实行排污许可制，推进排污权、用能权、用水权、碳排放权市场化交易。完善环境保护、节能减排约束性指标管理。完善中央生态环境保护督察制度。积极参与和引领应对气候变化等生态环保国际合作。

　　37.提升生态系统质量和稳定性。坚持山水林田湖草系统治理，构建以国家公园为主体的自然保护地体系。实施生物多样性保护重大工程。加强外来物种管控。强化河湖长制，加强大江大河和重要湖泊湿地生态保护治理，实施好长江十年禁渔。科学推进荒漠化、石漠化、水土流失综合治理，开展大规模国土绿化行动，推行林长制。推行草原森林河流湖泊休养生息，加强黑土地保护，健全耕地休耕轮作制度。加强全球气候变暖对我国承受力脆弱地区影响的观测，完善自然保护地、生态保护红线监管制度，开展生态系统保护成效监测评估。

　　38.全面提高资源利用效率。健全自然资源资产产权制度和法律法规，加强自然资源调查评价监测和确权登记，建立生态产品价值实现机制，完善市场化、多元化生态补偿，推进资源总量管理、科学配置、全面节约、循环利用。实施国家节水行动，建立水资源刚性约束制度。提高海洋资源、矿产资源开发保护水平。完善资源价格形成机制。推行垃圾分类和减量化、资源化。加快构建废旧物资循环利用体系。
　　十一、实行高水平对外开放，开拓合作共赢新局面
　　坚持实施更大范围、更宽领域、更深层次对外开放，依托我国大市场优势，促进国际合作，实现互利共赢。

　　39.建设更高水平开放型经济新体制。全面提高对外开放水平，推动贸易和投资自由化便利化，推进贸易创新发展，增强对外贸易综合竞争力。完善外商投资准入前国民待遇加负面清单管理制度，有序扩大服务业对外开放，依法保护外资企业合法权益，健全促进和保障境外投资的法律、政策和服务体系，坚定维护中国企业海外合法权益，实现高质量引进来和高水平走出去。完善自由贸易试验区布局，赋予其更大改革自主权，稳步推进海南自由贸易港建设，建设对外开放新高地。稳慎推进人民币国际化，坚持市场驱动和企业自主选择，营造以人民币自由使用为基础的新型互利合作关系。发挥好中国国际进口博览会等重要展会平台作用。

　　40.推动共建“一带一路”高质量发展。坚持共商共建共享原则，秉持绿色、开放、廉洁理念，深化务实合作，加强安全保障，促进共同发展。推进基础设施互联互通，拓展第三方市场合作。构筑互利共赢的产业链供应链合作体系，深化国际产能合作，扩大双向贸易和投资。坚持以企业为主体，以市场为导向，遵循国际惯例和债务可持续原则，健全多元化投融资体系。推进战略、规划、机制对接，加强政策、规则、标准联通。深化公共卫生、数字经济、绿色发展、科技教育合作，促进人文交流。

　　41.积极参与全球经济治理体系改革。坚持平等协商、互利共赢，推动二十国集团等发挥国际经济合作功能。维护多边贸易体制，积极参与世界贸易组织改革，推动完善更加公正合理的全球经济治理体系。积极参与多双边区域投资贸易合作机制，推动新兴领域经济治理规则制定，提高参与国际金融治理能力。实施自由贸易区提升战略，构建面向全球的高标准自由贸易区网络。
　　十二、改善人民生活品质，提高社会建设水平
　　坚持把实现好、维护好、发展好最广大人民根本利益作为发展的出发点和落脚点，尽力而为、量力而行，健全基本公共服务体系，完善共建共治共享的社会治理制度，扎实推动共同富裕，不断增强人民群众获得感、幸福感、安全感，促进人的全面发展和社会全面进步。

　　42.提高人民收入水平。坚持按劳分配为主体、多种分配方式并存，提高劳动报酬在初次分配中的比重，完善工资制度，健全工资合理增长机制，着力提高低收入群体收入，扩大中等收入群体。完善按要素分配政策制度，健全各类生产要素由市场决定报酬的机制，探索通过土地、资本等要素使用权、收益权增加中低收入群体要素收入。多渠道增加城乡居民财产性收入。完善再分配机制，加大税收、社保、转移支付等调节力度和精准性，合理调节过高收入，取缔非法收入。发挥第三次分配作用，发展慈善事业，改善收入和财富分配格局。

　　43.强化就业优先政策。千方百计稳定和扩大就业，坚持经济发展就业导向，扩大就业容量，提升就业质量，促进充分就业，保障劳动者待遇和权益。健全就业公共服务体系、劳动关系协调机制、终身职业技能培训制度。更加注重缓解结构性就业矛盾，加快提升劳动者技能素质，完善重点群体就业支持体系，统筹城乡就业政策体系。扩大公益性岗位安置，帮扶残疾人、零就业家庭成员就业。完善促进创业带动就业、多渠道灵活就业的保障制度，支持和规范发展新就业形态，健全就业需求调查和失业监测预警机制。

　　44.建设高质量教育体系。全面贯彻党的教育方针，坚持立德树人，加强师德师风建设，培养德智体美劳全面发展的社会主义建设者和接班人。健全学校家庭社会协同育人机制，提升教师教书育人能力素质，增强学生文明素养、社会责任意识、实践本领，重视青少年身体素质和心理健康教育。坚持教育公益性原则，深化教育改革，促进教育公平，推动义务教育均衡发展和城乡一体化，完善普惠性学前教育和特殊教育、专门教育保障机制，鼓励高中阶段学校多样化发展。加大人力资本投入，增强职业技术教育适应性，深化职普融通、产教融合、校企合作，探索中国特色学徒制，大力培养技术技能人才。提高高等教育质量，分类建设一流大学和一流学科，加快培养理工农医类专业紧缺人才。提高民族地区教育质量和水平，加大国家通用语言文字推广力度。支持和规范民办教育发展，规范校外培训机构。发挥在线教育优势，完善终身学习体系，建设学习型社会。

　　45.健全多层次社会保障体系。健全覆盖全民、统筹城乡、公平统一、可持续的多层次社会保障体系。推进社保转移接续，健全基本养老、基本医疗保险筹资和待遇调整机制。实现基本养老保险全国统筹，实施渐进式延迟法定退休年龄。发展多层次、多支柱养老保险体系。推动基本医疗保险、失业保险、工伤保险省级统筹，健全重大疾病医疗保险和救助制度，落实异地就医结算，稳步建立长期护理保险制度，积极发展商业医疗保险。健全灵活就业人员社保制度。健全退役军人工作体系和保障制度。健全分层分类的社会救助体系。坚持男女平等基本国策，保障妇女儿童合法权益。健全老年人、残疾人关爱服务体系和设施，完善帮扶残疾人、孤儿等社会福利制度。完善全国统一的社会保险公共服务平台。

　　46.全面推进健康中国建设。把保障人民健康放在优先发展的战略位置，坚持预防为主的方针，深入实施健康中国行动，完善国民健康促进政策，织牢国家公共卫生防护网，为人民提供全方位全周期健康服务。改革疾病预防控制体系，强化监测预警、风险评估、流行病学调查、检验检测、应急处置等职能。建立稳定的公共卫生事业投入机制，加强人才队伍建设，改善疾控基础条件，完善公共卫生服务项目，强化基层公共卫生体系。落实医疗机构公共卫生责任，创新医防协同机制。完善突发公共卫生事件监测预警处置机制，健全医疗救治、科技支撑、物资保障体系，提高应对突发公共卫生事件能力。坚持基本医疗卫生事业公益属性，深化医药卫生体制改革，加快优质医疗资源扩容和区域均衡布局，加快建设分级诊疗体系，加强公立医院建设和管理考核，推进国家组织药品和耗材集中采购使用改革，发展高端医疗设备。支持社会办医，推广远程医疗。坚持中西医并重，大力发展中医药事业。提升健康教育、慢病管理和残疾康复服务质量，重视精神卫生和心理健康。深入开展爱国卫生运动，促进全民养成文明健康生活方式。完善全民健身公共服务体系。加快发展健康产业。

　　47.实施积极应对人口老龄化国家战略。制定人口长期发展战略，优化生育政策，增强生育政策包容性，提高优生优育服务水平，发展普惠托育服务体系，降低生育、养育、教育成本，促进人口长期均衡发展，提高人口素质。积极开发老龄人力资源，发展银发经济。推动养老事业和养老产业协同发展，健全基本养老服务体系，发展普惠型养老服务和互助性养老，支持家庭承担养老功能，培育养老新业态，构建居家社区机构相协调、医养康养相结合的养老服务体系，健全养老服务综合监管制度。

　　48.加强和创新社会治理。完善社会治理体系，健全党组织领导的自治、法治、德治相结合的城乡基层治理体系，完善基层民主协商制度，实现政府治理同社会调节、居民自治良性互动，建设人人有责、人人尽责、人人享有的社会治理共同体。发挥群团组织和社会组织在社会治理中的作用，畅通和规范市场主体、新社会阶层、社会工作者和志愿者等参与社会治理的途径。推动社会治理重心向基层下移，向基层放权赋能，加强城乡社区治理和服务体系建设，减轻基层特别是村级组织负担，加强基层社会治理队伍建设，构建网格化管理、精细化服务、信息化支撑、开放共享的基层管理服务平台。加强和创新市域社会治理，推进市域社会治理现代化。
　　十三、统筹发展和安全，建设更高水平的平安中国
　　坚持总体国家安全观，实施国家安全战略，维护和塑造国家安全，统筹传统安全和非传统安全，把安全发展贯穿国家发展各领域和全过程，防范和化解影响我国现代化进程的各种风险，筑牢国家安全屏障。

　　49.加强国家安全体系和能力建设。完善集中统一、高效权威的国家安全领导体制，健全国家安全法治体系、战略体系、政策体系、人才体系和运行机制，完善重要领域国家安全立法、制度、政策。健全国家安全审查和监管制度，加强国家安全执法。加强国家安全宣传教育，增强全民国家安全意识，巩固国家安全人民防线。坚定维护国家政权安全、制度安全、意识形态安全，全面加强网络安全保障体系和能力建设。严密防范和严厉打击敌对势力渗透、破坏、颠覆、分裂活动。

　　50.确保国家经济安全。加强经济安全风险预警、防控机制和能力建设，实现重要产业、基础设施、战略资源、重大科技等关键领域安全可控。实施产业竞争力调查和评价工程，增强产业体系抗冲击能力。确保粮食安全，保障能源和战略性矿产资源安全。维护水利、电力、供水、油气、交通、通信、网络、金融等重要基础设施安全，提高水资源集约安全利用水平。维护金融安全，守住不发生系统性风险底线。确保生态安全，加强核安全监管，维护新型领域安全。构建海外利益保护和风险预警防范体系。

　　51.保障人民生命安全。坚持人民至上、生命至上，把保护人民生命安全摆在首位，全面提高公共安全保障能力。完善和落实安全生产责任制，加强安全生产监管执法，有效遏制危险化学品、矿山、建筑施工、交通等重特大安全事故。强化生物安全保护，提高食品药品等关系人民健康产品和服务的安全保障水平。提升洪涝干旱、森林草原火灾、地质灾害、地震等自然灾害防御工程标准，加快江河控制性工程建设，加快病险水库除险加固，全面推进堤防和蓄滞洪区建设。完善国家应急管理体系，加强应急物资保障体系建设，发展巨灾保险，提高防灾、减灾、抗灾、救灾能力。

　　52.维护社会稳定和安全。正确处理新形势下人民内部矛盾，坚持和发展新时代“枫桥经验”，畅通和规范群众诉求表达、利益协调、权益保障通道，完善信访制度，完善各类调解联动工作体系，构建源头防控、排查梳理、纠纷化解、应急处置的社会矛盾综合治理机制。健全社会心理服务体系和危机干预机制。坚持专群结合、群防群治，加强社会治安防控体系建设，坚决防范和打击暴力恐怖、黑恶势力、新型网络犯罪和跨国犯罪，保持社会和谐稳定。
　　十四、加快国防和军队现代化，实现富国和强军相统一
　　贯彻习近平强军思想，贯彻新时代军事战略方针，坚持党对人民军队的绝对领导，坚持政治建军、改革强军、科技强军、人才强军、依法治军，加快机械化信息化智能化融合发展，全面加强练兵备战，提高捍卫国家主权、安全、发展利益的战略能力，确保二〇二七年实现建军百年奋斗目标。

　　53.提高国防和军队现代化质量效益。加快军事理论现代化，与时俱进创新战争和战略指导，健全新时代军事战略体系，发展先进作战理论。加快军队组织形态现代化，深化国防和军队改革，推进军事管理革命，加快军兵种和武警部队转型建设，壮大战略力量和新域新质作战力量，打造高水平战略威慑和联合作战体系，加强军事力量联合训练、联合保障、联合运用。加快军事人员现代化，贯彻新时代军事教育方针，完善三位一体新型军事人才培养体系，锻造高素质专业化军事人才方阵。加快武器装备现代化，聚力国防科技自主创新、原始创新，加速战略性前沿性颠覆性技术发展，加速武器装备升级换代和智能化武器装备发展。

　　54.促进国防实力和经济实力同步提升。同国家现代化发展相协调，搞好战略层面筹划，深化资源要素共享，强化政策制度协调，构建一体化国家战略体系和能力。推动重点区域、重点领域、新兴领域协调发展，集中力量实施国防领域重大工程。优化国防科技工业布局，加快标准化通用化进程。完善国防动员体系，健全强边固防机制，强化全民国防教育，巩固军政军民团结。
　　十五、全党全国各族人民团结起来，为实现“十四五”规划和二〇三五年远景目标而奋斗
　　实现“十四五”规划和二〇三五年远景目标，必须坚持党的全面领导，充分调动一切积极因素，广泛团结一切可以团结的力量，形成推动发展的强大合力。

　　55.加强党中央集中统一领导。贯彻党把方向、谋大局、定政策、促改革的要求，推动全党深入学习贯彻习近平新时代中国特色社会主义思想，增强“四个意识”、坚定“四个自信”、做到“两个维护”，完善上下贯通、执行有力的组织体系，确保党中央决策部署有效落实。落实全面从严治党主体责任、监督责任，提高党的建设质量。深入总结和学习运用中国共产党一百年的宝贵经验，教育引导广大党员、干部坚持共产主义远大理想和中国特色社会主义共同理想，不忘初心、牢记使命，为党和人民事业不懈奋斗。全面贯彻新时代党的组织路线，加强干部队伍建设，落实好干部标准，提高各级领导班子和干部适应新时代新要求抓改革、促发展、保稳定水平和专业化能力，加强对敢担当善作为干部的激励保护，以正确用人导向引领干事创业导向。完善人才工作体系，培养造就大批德才兼备的高素质人才。把严的主基调长期坚持下去，不断增强党自我净化、自我完善、自我革新、自我提高能力。锲而不舍落实中央八项规定精神，持续纠治形式主义、官僚主义，切实为基层减负。完善党和国家监督体系，加强政治监督，强化对公权力运行的制约和监督。坚持无禁区、全覆盖、零容忍，一体推进不敢腐、不能腐、不想腐，营造风清气正的良好政治生态。

　　56.推进社会主义政治建设。坚持党的领导、人民当家作主、依法治国有机统一，推进中国特色社会主义政治制度自我完善和发展。坚持和完善人民代表大会制度，加强人大对“一府一委两院”的监督，保障人民依法通过各种途径和形式管理国家事务、管理经济文化事业、管理社会事务。坚持和完善中国共产党领导的多党合作和政治协商制度，加强人民政协专门协商机构建设，发挥社会主义协商民主独特优势，提高建言资政和凝聚共识水平。坚持和完善民族区域自治制度，全面贯彻党的民族政策，铸牢中华民族共同体意识，促进各民族共同团结奋斗、共同繁荣发展。全面贯彻党的宗教工作基本方针，积极引导宗教与社会主义社会相适应。健全基层群众自治制度，增强群众自我管理、自我服务、自我教育、自我监督实效。发挥工会、共青团、妇联等人民团体作用，把各自联系的群众紧紧凝聚在党的周围。完善大统战工作格局，促进政党关系、民族关系、宗教关系、阶层关系、海内外同胞关系和谐，巩固和发展大团结大联合局面。全面贯彻党的侨务政策，凝聚侨心、服务大局。坚持法治国家、法治政府、法治社会一体建设，完善以[宪法](https://www.pkulaw.com/chl/7c7e81f43957c58bbdfb.html)为核心的中国特色社会主义法律体系，加强重点领域、新兴领域、涉外领域立法，提高依法行政水平，完善监察权、审判权、检察权运行和监督机制，促进司法公正，深入开展法治宣传教育，有效发挥法治固根本、稳预期、利长远的保障作用，推进法治中国建设。促进人权事业全面发展。

　　57.保持香港、澳门长期繁荣稳定。全面准确贯彻“一国两制”、“港人治港”、“澳人治澳”、高度自治的方针，坚持依法治港治澳，维护[宪法](https://www.pkulaw.com/chl/7c7e81f43957c58bbdfb.html)和基本法确定的特别行政区宪制秩序，落实中央对特别行政区全面管治权，落实特别行政区维护国家安全的法律制度和执行机制，维护国家主权、安全、发展利益和特别行政区社会大局稳定。支持特别行政区巩固提升竞争优势，建设国际创新科技中心，打造“一带一路”功能平台，实现经济多元可持续发展。支持香港、澳门更好融入国家发展大局，高质量建设粤港澳大湾区，完善便利港澳居民在内地发展政策措施。增强港澳同胞国家意识和爱国精神。支持香港、澳门同各国各地区开展交流合作。坚决防范和遏制外部势力干预港澳事务。

　　58.推进两岸关系和平发展和祖国统一。坚持一个中国原则和“九二共识”，以两岸同胞福祉为依归，推动两岸关系和平发展、融合发展，加强两岸产业合作，打造两岸共同市场，壮大中华民族经济，共同弘扬中华文化。完善保障台湾同胞福祉和在大陆享受同等待遇的制度和政策，支持台商台企参与“一带一路”建设和国家区域协调发展战略，支持符合条件的台资企业在大陆上市，支持福建探索海峡两岸融合发展新路。加强两岸基层和青少年交流。高度警惕和坚决遏制“***”分裂活动。

　　59.积极营造良好外部环境。高举和平、发展、合作、共赢旗帜，坚持独立自主的和平外交政策，推进各领域各层级对外交往，推动构建新型国际关系和人类命运共同体。推进大国协调和合作，深化同周边国家关系，加强同发展中国家团结合作，积极发展全球伙伴关系。坚持多边主义和共商共建共享原则，积极参与全球治理体系改革和建设，加强涉外法治体系建设，加强国际法运用，维护以联合国为核心的国际体系和以国际法为基础的国际秩序，共同应对全球性挑战。积极参与重大传染病防控国际合作，推动构建人类卫生健康共同体。

　　60.健全规划制定和落实机制。按照本次全会精神，制定国家和地方“十四五”规划纲要和专项规划，形成定位准确、边界清晰、功能互补、统一衔接的国家规划体系。健全政策协调和工作协同机制，完善规划实施监测评估机制，确保党中央关于“十四五”发展的决策部署落到实处。
　　实现“十四五”规划和二〇三五年远景目标，意义重大，任务艰巨，前景光明。全党全国各族人民要紧密团结在以习近平同志为核心的党中央周围，同心同德，顽强奋斗，夺取全面建设社会主义现代化国家新胜利！

© 北大法宝：（[www.pkulaw.com](https://www.pkulaw.com/)) 专业提供法律信息、法学知识和法律软件领域各类解决方案。北大法宝为您提供丰富的参考资料，正式引用法规条文时请与标准文本核对。
欢迎查看所有[产品和服务](https://www.pkulaw.com/helps/2.html)。法宝快讯：[如何快速找到您需要的检索结果？](https://www.pkulaw.com/helps/70.html)[法宝](https://www.pkulaw.com/helps/69.html)V6有何新特色？

扫描二维码阅读原文

**原文链接：**<https://www.pkulaw.com/chl/62a4170c41f8f103bdfb.html>
